# Tumor suppressive microRNA-218 inhibits cancer cell migration and invasion through targeting laminin-332 in head and neck squamous cell carcinoma

**DOI:** 10.18632/oncotarget.709

**Published:** 2012-11-07

**Authors:** Takashi Kinoshita, Toyoyuki Hanazawa, Nijiro Nohata, Naoko Kikkawa, Hideki Enokida, Hirofumi Yoshino, Takeshi Yamasaki, Hideo Hidaka, Masayuki Nakagawa, Yoshitaka Okamoto, Naohiko Seki

**Affiliations:** ^1^ Department of Functional Genomics, Chiba University Graduate School of Medicine, 1-8-1 Inohana Chuo-ku, Chiba, Japan; ^2^ Department of Otorhinolaryngology / Head and Neck Surgery, Chiba University Graduate School of Medicine, Chiba, Japan; ^3^ Department of Urology, Graduate School of Medical and Dental Sciences, Kagoshima University, Kagoshima, Japan

**Keywords:** microRNA, miR-218, tumor suppressor, focal adhesion, laminin-332, head and neck squamous cell carcinoma

## Abstract

Recent our microRNA (miRNA) expression signature revealed that expression of microRNA-218 (miR-218) was reduced in cancer tissues, suggesting a candidate of tumor suppressor in head and neck squamous cell carcinoma (HNSCC). The aim of this study was to investigate the functional significance of miR-218 and its mediated moleculer pathways in HNSCC. Restoration of miR-218 in cancer cells led to significant inhibition of cell migration and invasion activities in HNSCC cell lines (FaDu and SAS). Genome-wide gene expression analysis of miR-218 transfectants and in silico database analysis showed that focal adhesion pathway was a promising candidate of miR-218 target pathways. The laminins are an important and biologically active part of the basal lamina, the function of that are various such as influencing cell differentiation, migration and adhesion as well as proliferation and cell survival. Interestingly, all components of laminin-332 (LAMA3, LAMB3 and LAMC2) are listed on the candidate genes in focal adhesion pathway. Furthermore, we focused on LAMB3 which has a miR-218 target site and gene expression studies and luciferase reporter assays showed that LAMB3 was directly regulated by miR-218. Silencing study of LAMB3 demonstrated significant inhibition of cell migration and invasion. In clinical specimens with HNSCC, the expression levels of laminin-332 were significantly upregulated in cancer tissues compared to adjacent non-cancerous tissues. Our analysis data showed that tumor suppressive miR-218 contributes to cancer cell migration and invasion through regulating focal adhesion pathway, especially laminin-332. Tumor suppressive miRNA-mediated novel cancer pathways provide new insights into the potential mechanisms of HNSCC oncogenesis.

## INTRODUCTION

Head and neck squamous cell carcinoma (HNSCC) is the sixth most common cancer worldwide and approximately 650,000 new cases occur and 350,000 patients dying from HNSCC annually [1]. Despite of considerable advances in multimodality therapy including surgery, radiation therapy, and chemotherapy, the overall survival rate for patients with this type of cancer is among the lowest of all major cancer types and has not improved during recent decades [2]. Local tumor recurrence and distant metastasis after conventional therapy appear to be major contributing factors for restricted survival of HNSCC patients [2]. Therefore, understanding the molecular metastatic pathways underlying HNSCC would help to improve diagnosis, approaches to therapy, and prevention of the disease.

The discovery of non-coding RNA in human genome is a topic in post genome sequencing era [3]. The reconstructing of the genome-wide study, including non-coding RNA is necessary for cancer research at present. microRNAs (miRNAs) are most characterized among non-coding RNAs. miRNAs are a class of small non-coding RNA molecules consisting of 19–22 nucleotides that play important roles in a variety of biological processes, including development, differentiation, apoptosis and cell proliferation [4].

A growing body of evidence indicated that miRNAs also contributed to the initiation and development of various types of cancers [5, 6]. Many human cancers have aberrant expression of miRNAs, which can function either as tumor suppressors or oncogenes [6]. miRNAs are unique in their ability to regulate many protein-coding genes. Bioinformatics predictions indicate that miRNAs regulate more than 30% of the protein coding genes in human genome [4]. In cancer pathways, normal regulatory mechanisms are disrupted by altered expression of tumor suppressive or oncogenic miRNAs.

We previously identified tumor suppressive miRNAs based on miRNA expression signatures of various types of cancer, such as hypopharyngeal, maxillary sinus, esophageal, and lung SCCs, and renal cell carcinoma and bladder cancer [7-13]. We hypothesize that normal regulatory miRNA-mRNA molecular mechanisms are disrupted by aberrant expression of tumor suppressive or oncogenic miRNAs in cancer cells. Therefore, we have sequentially identified tumor suppressive miRNAs regulated novel cancer pathways [7, 8, 14-20].

According to our HNSCC miRNA expression signatures, *microRNA-218* (*miR-218*) was significantly reduced in cancer tissues [7, 8]. The downregulation of *miR-218* was also reported in several cancers and its targeting cancer-related genes were identified [21-26]. The aim of the study was to investigate the functional significance of *miR-218* and identify its regulating molecular pathways in HNSCC cells. Genome-wide gene expression analysis of *miR-218* transfectant and *in silico* database analysis showed that focal adhesion pathway was a promising candidate of *miR-218* target pathway. The laminins are an important and biologically active part of the basal lamina, influencing cell differentiation, migration and adhesion as well as proliferation and cell survival. Interestingly, all components of laminin-332 (*LAMA3*, *LAMB3* and *LAMC2*) are listed on the candidate genes in focal adhesion pathway. Furthermore, we focused on *LAMB3* which has a *miR-218* target site and gene expression studies and luciferase reporter assays showed that *LAMB3* was directly regulated by *miR-218*. We also investigated functional significance of *LAMB3* in HNSCC and its regulated novel molecular pathways. Tumor suppressive *miR-218*-mediated novel cancer pathways provide new insights into the potential mechanisms of HNSCC oncogenesis and metastasis.

## RESULTS

### Effects of miR-218 transfection on proliferation, migration and invasion in HNSCC cells, FaDu and SAS

**Figure 1:**
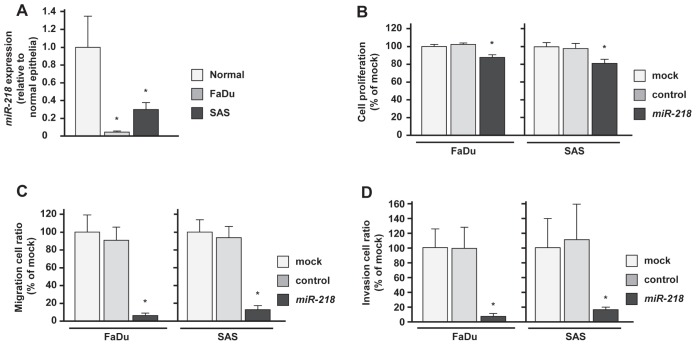
Effects of miR-218 transfection on HNSCC cell lines, FaDu and SAS (A)Real-time RT-PCR showed that expression levels of *miR-218* was significantly lower in both cell lines, FaDu and SAS than in normal epithelial specimens. (B)Suppression of FaDu and SAS cell proliferation after transfection (72h) with *miR-218* (10nM) was determined by XTT assay. (C) Suppression of FaDu and SAS cell migration activity after transfection (72h) with *miR-218* (10nM) was determined by migration assay. (D) Suppression of FaDu and SAS cell invasion activity after transfection (72h) with *miR-218* (10nM) was determined by Matrigel invasion assay. *p < 0.0167.

The expression levels of *miR-218* in HNSCC cells (FaDu and SAS) were significantly downregulated compared with those in normal epithelial tissues (Figure 1A). This is why we used these cell lines to investigate functional analysis of *miR-218* in this study. To investigate the tumor suppressive roles of *miR-218*, we conducted gain-of-function analysis using mature miRNA transient transfection.

The XTT assay showed that the cell proliferation in *miR-218* transfectants was reduced to approximately 85% of mock in FaDu and SAS (Figure 1B). The migration assay revealed that the number of migrated cells was significantly decreased in *miR-218* transfectants compared with mock and miR-control transfectants (% of migrated cells relative to mock; FaDu, 3.9 ± 2.3, p < 0.0167; SAS, 11.7 ± 4.5, p < 0.0167; Figure 1C). The Matrigel invasion assay also showed significant decrease in the number of invaded cells in *miR-218* transfectants (% of invaded cells relative to mock; FaDu, 3.1 ± 3.0, p < 0.0167; SAS, 17.1 ± 9.7, p < 0.0167; Figure 1D).

### Identification of molecular pathways regulated by miR-218 in HNSCC

To find the target genes of *miR-218* in HNSCC cells, we performed gene expression profiling using *miR-218*-transfected FaDu and SAS in comparison with the miR-control transfectants. When compared with the expression of miR-control transfectants, 525 genes were downregulated (log2 ratio < -0.5) in FaDu transfected with *miR-218* and 831 genes in SAS (Supplementary table 1 and 2). Entries from the microarray data were approved by the Gene Expression Omnibus (GEO), and were assigned GEO accession numbers GSE37119. These genes were assigned to Kyoto Encyclopedia of Genes and Genomes (KEGG) annotations using singular enrichment analysis of GeneCodis [27, 28] and significantly enriched annotations in FaDu and SAS were listed (Table 1 and Table 2).

**Table 1:** Significantly enriched annotations regulated by miR-218 in FaDu

Number of Genes	p Value	Annotations
18	2.34E-08	Focal adhesion
16	1.07E-11	Systemic lupus erythematosus
15	1.51E-03	Pathways in cancer
13	2.82E-04	Regulation of actin cytoskeleton
10	1.97E-03	Protein processing in endoplasmic reticulum
10	2.87E-02	MAPK signaling pathway
9	1.70E-02	Endocytosis
8	1.94E-03	Amoebiasis
8	9.35E-03	Hepatitis C
8	1.36E-02	Wnt signaling pathway
8	3.36E-02	Chemokine signaling pathway
7	3.25E-04	Mineral absorption
7	3.24E-03	Small cell lung cancer
7	1.44E-02	Leukocyte transendothelial migration
6	1.46E-02	ECM-receptor interaction
6	4.70E-02	Toxoplasmosis
5	2.52E-02	Adipocytokine signaling pathway
5	2.52E-02	Long-term potentiation
5	2.54E-02	Bacterial invasion of epithelial cells
5	2.89E-02	Arrhythmogenic right ventricular cardiomyopathy
4	9.16E-03	Thyroid cancer
4	2.54E-02	Bladder cancer
4	4.08E-02	Endometrial cancer
4	4.18E-02	Non-small cell lung cancer
3	4.02E-02	beta-Alanine metabolism
2	4.92E-02	Valine, leucine and isoleucine biosynthesis

**Table 2:** Significantly enriched annotations regulated by miR-218 in SAS

Number of Genes	p Value	Annotations
21	2.05E-04	Pathways in cancer
17	1.45E-04	Focal adhesion
15	4.58E-04	Endocytosis
14	2.79E-03	Regulation of actin cytoskeleton
13	8.44E-04	Protein processing in endoplasmic reticulum
11	1.14E-04	ECM-receptor interaction
10	4.30E-03	Lysosome
10	4.37E-02	Chemokine signaling pathway
9	4.89E-03	Amoebiasis
8	1.22E-04	Bladder cancer
8	2.53E-03	Pancreatic cancer
8	6.78E-03	Apoptosis
8	3.94E-02	Glutamatergic synapse
8	4.36E-02	Tight junction
7	7.05E-03	Adipocytokine signaling pathway
7	2.18E-02	Small cell lung cancer
6	7.05E-03	Lysine degradation
6	8.15E-03	Mineral absorption
6	2.82E-02	p53 signaling pathway
6	3.33E-02	PPAR signaling pathway
6	3.38E-02	Adherens junction
5	3.95E-02	Non-small cell lung cancer
5	3.95E-02	Vibrio cholerae infection
4	2.18E-02	beta-Alanine metabolism

We focused on the focal adhesion pathway because this pathway can be implicated in cancer cell migration and invasion and can be a promising candidate of *miR-218* target pathway. The genes that were annotated as focal adhesion pathway were then analyzed using *in silico* target prediction databases (miRWalk and TargetScan), generating a list of candidate target genes of *miR-218* (Table 3). Interestingly, all three subunits of laminin-332 (*LAMA3*, *LAMB3* and *LAMC2*) are all listed on these candidates. Therefore, we focused on laminin-332 as the target of *miR-218* and further studied.

**Table 3:** Candidate target genes of miR-218 in the focal adhesion pathway

Gene symbol	Log2 ratio (miR-218/miR-control)			Putative miR-218 target site	
	FaDu	SAS	Average	miR Walk	Target Scan
CAV2	−1.97	−1.73	−1.85	+	+
PPP1CB	−1.98	−1.65	−1.82	+	+
LAMC2	−1.51	−0.96	−1.24	−	−
ACTN1	−1.13	−1.27	−1.20	+	+
THBS2	−0.60	−1.61	−1.11	−	−
LAMB3	−1.25	−0.90	−1.08	+	+
CAV1	−1.20	−0.95	−1.08	−	−
EGFR	−0.85	−1.19	−1.02	+	+
VEGFC	−0.90	−1.03	−0.97	−	−
PPP1CC	−0.87	−0.97	−0.92	+	+
COL5A2	0.93	−2.66	−0.87	−	−
CCND2	−1.06	−0.62	−0.84	−	+
VASP	−0.82	−0.72	−0.77	−	−
CAV3	−0.87	−0.66	−0.77	+	+
THBS1	−0.66	−0.86	−0.76	+	+
COL4A2	−0.72	−0.58	−0.65	−	−
ACTN4	−0.69	−0.57	−0.63	+	+
ITGA3	−0.69	−0.53	−0.61	−	−
CCND3	−0.76	−0.37	−0.57	+	+
SHC1	−0.64	−0.45	−0.55	+	+
ITGB4	−0.61	−0.43	−0.52	−	+
PXN	−0.67	−0.33	−0.50	+	+
LAMC1	−0.59	−0.35	−0.47	+	+
VEGFA	−0.19	−0.63	−0.41	−	−
ERBB2	−0.06	−0.62	−0.34	−	−
LAMA3	−0.76	0.08	−0.34	−	−
FN1	−0.11	−0.54	−0.33	−	+
MAPK1	−0.55	−0.09	−0.32	−	−
FLNB	−0.63	0.30	−0.17	−	−

### Expression levels of miR-218 and laminin-332 in HNSCC clinical specimens

**Figure 2:**
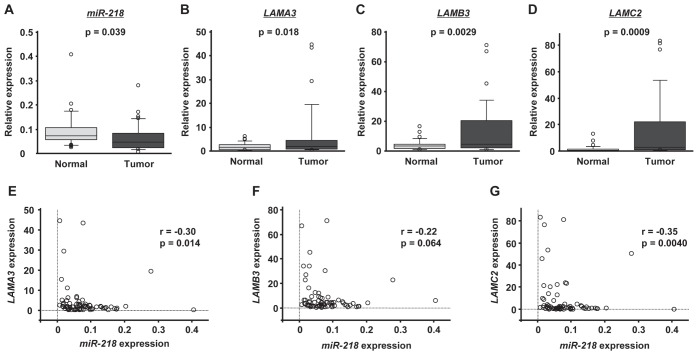
The expression levels of miR-218 and laminin-332 in HNSCC clinical specimens The expression levels of *miR-218* (A), *LAMA3* (B), *LAMB3* (C) and *LAMC2* (D) in tumor tissues and adjacent normal epithelium tissues of 35 HNSCC patients were determined by qRT-PCR. *RNU48* and *GUSB* were used as internal controls. The correlated expression of *miR-218* and laminin-33*2* was determined in HNSCC specimens. The correlation coefficient indicates that *miR-218* expression was highly correlated with that of laminin-332, *LAMA3* (E), *LAMB3* (F) and *LAMC2* (G).

We performed qRT-PCR to compare the expression levels of *miR-218*, *LAMA3*, *LAMB3* and *LAMC2* in clinical HNSCC specimens. Clinical information for the 35 patients is shown in Table 4. The expression level of *miR-218* was significantly downregulated in tumor tissues compared with adjacent normal tissues (p = 0.039; Figure 2A), whereas *LAMA3*, *LAMB3* and *LAMC2* were upregulated in tumor tissues (P = 0.018, p = 0.0029 and p = 0.0009, respectively; Figure 2B - 2D). Interestingly, *LAMA3* and *LAMC2* expression was significantly inversely correlated with *miR-218* expression (r = −0.30, p = 0.014; and r = −0.35, p = 0.0040, respectively; Figure 2E and 2G). Although the expression of *LAMB3* tended to inversely correlate with that of *miR-218*, statistical significance was not observed (r = −0.22, p = 0.064; Figure 2F).

**Table 4:** Clinical features for 35 patients with HNSCC

No.	Age	Sex	Site	T	N	M	Stage	Differentiaion
1	68	M	hypopharynx	4a	0	0	IVA	moderately
2	73	M	hypopharynx	3	1	0	III	poorly
3	66	M	hypopharynx	2	2c	0	IVA	moderately
4	68	M	hypopharynx	2	2b	0	IVA	poorly
5	65	M	hypopharynx	1	2b	0	IVA	moderately
6	71	M	hypopharynx	2	2b	0	IVA	poorly
7	64	F	hypopharynx	4a	0	0	IVA	well
8	64	M	hypopharynx	2	0	0	II	moderately
9	55	M	hypopharynx	3	2b	0	IVA	moderately
10	61	F	hypopharynx	4a	2c	0	IVA	poorly
11	66	M	hypopharynx	4a	2c	0	IVA	well
12	58	F	hypopharynx	4a	2c	0	IVA	moderately
13	52	M	hypopharynx	4a	1	1	IVC	moderately
14	74	M	hypopharynx	4a	2c	0	IVA	poorly
15	64	M	hypopharynx	2	0	0	II	moderately
16	45	M	hypopharynx	4a	2c	0	IVA	moderately
17	64	F	hypopharynx	4a	1	0	IVA	poorly
18	79	M	hypopharynx	2	2c	0	IVA	moderately
19	69	M	hypopharynx	2	2b	0	IVA	poorly
20	61	M	hypopharynx	3	0	0	III	moderately
21	77	M	gingiva	2	0	0	II	moderately
22	65	M	oral floor	4a	1	0	IVA	moderately
23	63	F	oral floor	2	2b	0	IVA	moderately
24	68	M	tongue	2	0	0	II	moderate
25	66	M	tongue	2	0	0	II	moderate
26	76	F	tongue	1	0	0	I	well
27	69	M	tongue	1	0	0	I	well
28	73	F	tongue	1	0	0	I	well
29	67	M	tongue	4a	2c	0	IVA	moderately
30	36	F	tongue	3	1	0	III	moderately
31	67	M	tongue	3	0	0	III	moderately
32	51	M	tongue	1	0	0	I	well
33	70	M	tongue	4a	0	0	IVA	moderately
34	71	M	tongue	1	0	0	I	well
35	60	F	tongue	2	2b	0	IVA	well

### Regulation of laminin-332 expression by miR-218

**Figure 3:**
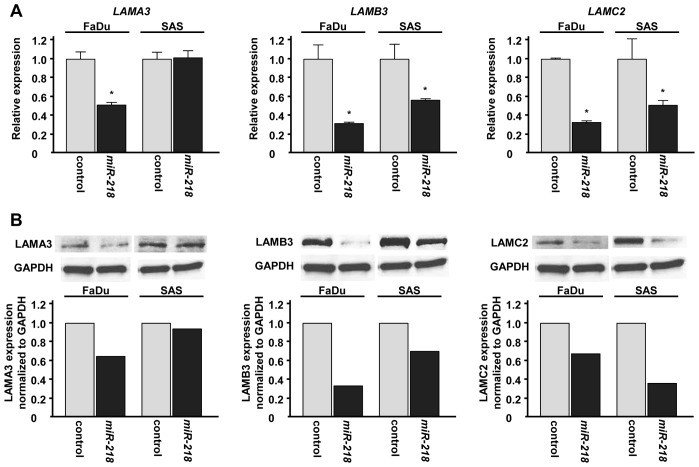
Laminin-332 expression was suppressed by miR-218 transfection at both the mRNA and protein levels in HNSCC cell lines (A) mRNA expression of *LAMA3*, *LAMB3* and *LAMC2* as revealed by qRT-PCR 48 h after transfection with 10 nM of *miR-218*. *GUSB* was used as internal controls. *p< 0.05. (B) Protein expression of LAMA3, LAMB3 and LAMC2 as revealed by western blot analysis 48 h after transfection with 10 nM of *miR-218*. GAPDH was used as loading controls. The expression ratio of LAMA3, LAMB3 and LAMC2 were evaluated using ImageJ software.

To investigate the effect of *miR-218* transfection on the expression levels of laminin-332 (*LAMA3*, *LAMB3* and *LAMC2*), we performed qRT-PCR and Western blotting using FaDu and SAS. The mRNA expression levels of *LAMB3* and *LAMC2* were significantly decreased in *miR-218* transfectants compared with miR-control transfectants (Figure 3A). Although restoration of *miR-218* significantly suppressed *LAMA3* expression in FaDu, no significant downregulation of *LAMA3* was observed in SAS (Figure 3A). Western blotting demonstrated similar effect of *miR-218* on protein expression levels of laminins-332 (Figure 3B).

**Figure 4:**
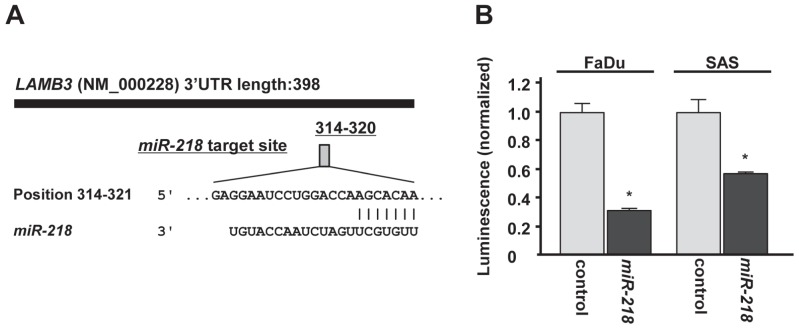
miR-218 directly regulates LAMB3 by luciferase reporter assay (A)Putative *miR-218* binding site in the 3'UTR of *LAMB3* mRNA was identified with the TargetScan database. (B) Luciferase reporter assay was performed using the vector encoding partial sequences of 3'UTR which contained the putative *miR-218* target site. The vector (10 ng) and *miR-218* or *miR-control* (10 nM) were cotransfected into FaDu and SAS cell lines. Renila luciferase activity was measured after 24h transfection. The results were normalized by firefly luciferase values. *p < 0.05.

The target prediction databases indicated one putative target site in the 3' untranslated region (3'UTR) of *LAMB3* (Figure 4A). To determine whether *LAMB3* mRNA had a functional target site, we performed luciferase reporter assay. The luminescence intensity was significantly reduced by transfection of *miR-218* compared with miR-control transfection (Figure 4B).

### Effect of LAMB3 silencing on cell proliferation, migration and invasion activities of HNSCC cell lines

**Figure 5:**
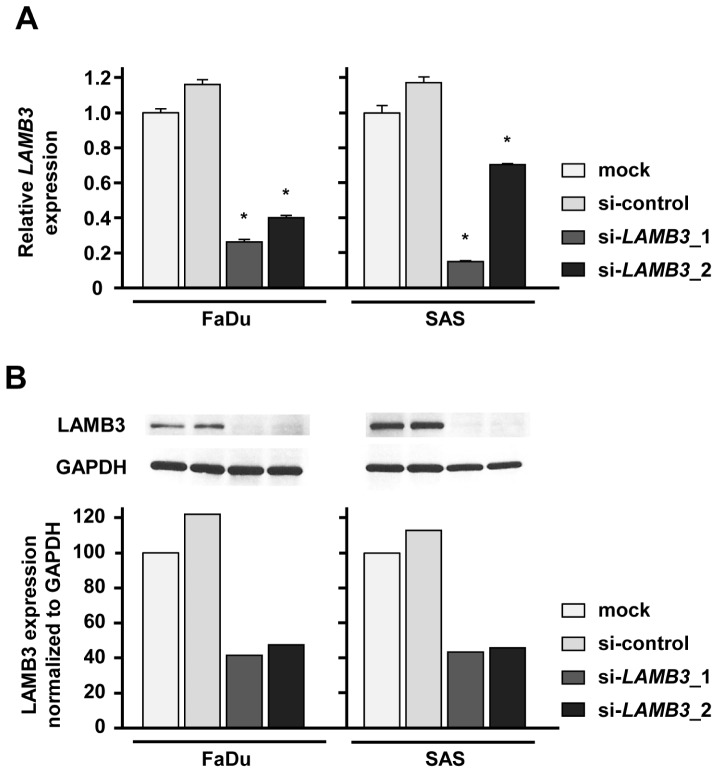
LAMB3 expression was suppressed by si-LAMB3 transfection at both the mRNA and protein levels in HNSCC cell lines (A) mRNA expression of *LAMB3* as revealed by real-time qRT-PCR 48 h after transfection with 10 nM of si-*LAMB3*. *p < 0.0083. (B) Protein expression of LAMB3 as revealed by western blot analysis 48 h after transfection with 10 nM of si-*LAMB3*. GAPDH was used as loading controls. The expression ratio of LAMB3 was evaluated using ImageJ software.

A loss-of-function assay using siRNA analysis was performed to examine the function of *LAMB3* in cancer cells. The expression levels of *LAMB3* mRNA and LAMB3 protein were repressed by si-*LAMB3* in FaDu and SAS (Figure 5A and 5B).

**Figure 6:**
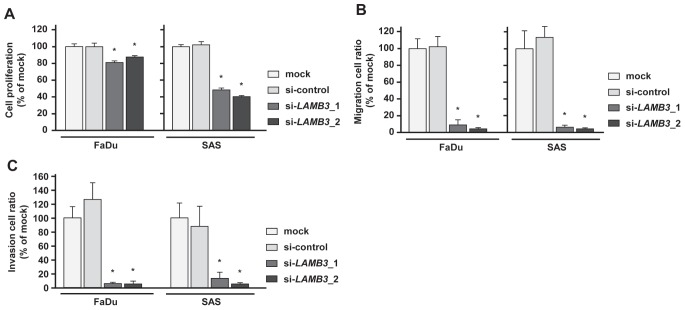
Effects of LAMB3 knockdown by si-LAMB3 transfection on HNSCC cell lines, FaDu and SAS (A) Cell proliferation activities as revealed by XTT assay in HNSCC cell lines, FaDu and SAS. *p < 0.0083 (B) Cell migration activities (migration assay) of HNSCC cells. *p < 0.0083 (C) Cell invasion activities (Matrigel invasion assay) of HNSCC cells. *p < 0.0083

The XTT assay revealed that cell proliferation was significantly repressed in FaDu and SAS 72 h after si-*LAMB3* transfection compared with mock and si-control (Figure 6A). Migration assays revealed that the number of migrated cells was significantly smaller in si-*LAMB3* transfectants compared with mock and si-controls (Figure 6B), and Matrigel invasion assays also showed that the number of invaded cells was significantly smaller in si-*LAMB3* transfectants in FaDu and SAS (Figure 6C).

### Effect of LAMB3 silencing on molecular pathways

**Figure 7:**
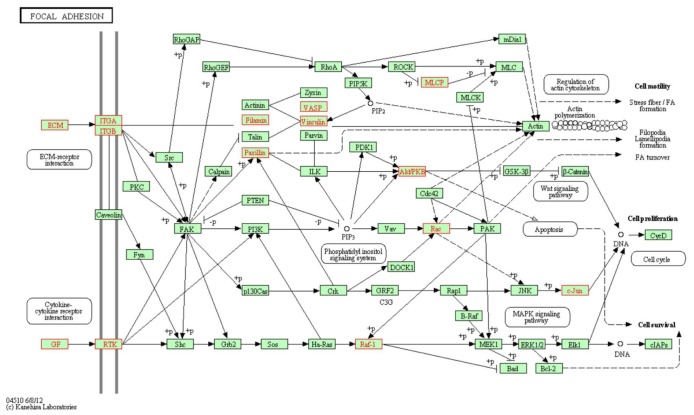
Putative LAMB3 regulated genes in “focal adhesion pathway” Putative *LAMB3* regulated genes in “focal adhesion pathway” from Kyoto Encyclopedia of Genes and Genomes (KEGG). The putative *LAMB3* regulated genes (highlighted in red) as defined by KEGG pathway and determined through GENECODIS analysis.

To investigate molecular pathways regulated by *LAMB3*, a genome-wide gene expression analysis was performed comparing the expression of si-*LAMB3* transfectants with that of si-control transfectants in FaDu. A total of 852 genes were downregulated and 381 genes were upregulated in si-*LAMB3* transfectants (Supplementary Table 3 and 4). We assigned the downregulated genes to KEGG annotations using singular enrichment analysis of GeneCodis [27, 28] and significantly enriched annotations were listed in table 5. We focused on the Focal adhesion pathway and the genes categorized to this pathway were listed in Table 6 and highlighted in KEGG map (Figure 7). Among them, several integrin signaling downstream molecules (*JUN*, *RAC1* and *PXN*) were included.

**Table 5:** Pathway analysis of downregulated genes in si-LAMB3 transfected FaDu

Nunber of genes	P value	Annotations
20	6.64E-04	Pathways in cancer
19	4.90E-06	Focal adhesion
16	9.86E-09	Systemic lupus erythematosus
14	3.97E-04	Protein processing in endoplasmic reticulum
13	1.23E-04	Lysosome
13	8.46E-04	Purine metabolism
13	2.96E-03	Chemokine signaling pathway
13	7.78E-03	Regulation of actin cytoskeleton
11	7.38E-04	Oocyte meiosis
9	1.41E-03	ECM-receptor interaction
9	1.41E-03	Small cell lung cancer
8	4.27E-04	Mineral absorption
8	2.02E-03	Pancreatic cancer
8	9.76E-03	Pyrimidine metabolism
7	8.91E-03	Renal cell carcinoma
6	4.60E-03	Glutathione metabolism
4	9.93E-03	Biosynthesis of unsaturated fatty acids

**Table 6:** Downregulated genes in si-LAMB3 transfectants in the focal adhesion pathway

Gene symbol	Gene name	Log2 ratio
LAMB3	laminin, beta 3	−2.50
VASP	vasodilator-stimulated phosphoprotein	−2.23
LAMC2	laminin, gamma 2	−1.80
JUN	jun proto-oncogene	−1.56
ITGAV	integrin, alpha V (vitronectin receptor, alpha polypeptide, antigen CD51)	−1.17
IGF1R	insulin-like growth factor 1 receptor	−1.08
ITGB1	integrin, beta 1 (fibronectin receptor, beta polypeptide, antigen CD29 includes MDF2, MSK12)	−0.96
RAF1	v-raf-1 murine leukemia viral oncogene homolog 1	−0.91
VEGFC	vascular endothelial growth factor C	−0.90
LAMC1	laminin, gamma 1 (formerly LAMB2)	−0.84
LAMA3	laminin, alpha 3	−0.82
FLNB	filamin B, beta	−0.66
ITGA3	integrin, alpha 3 (antigen CD49C, alpha 3 subunit of VLA-3 receptor)	−0.65
PXN	paxillin	−0.62
VCL	vinculin	−0.61
MET	met proto-oncogene (hepatocyte growth factor receptor)	−0.58
RAC1	ras-related C3 botulinum toxin substrate 1 (rho family, small GTP binding protein Rac1)	−0.58
AKT2	v-akt murine thymoma viral oncogene homolog 2	−0.55
PPP1CC	protein phosphatase 1, catalytic subunit, gamma isozyme	−0.50

### DISCUSSION

Despite of the considerable advances in cancer treatments, the overall survival rate of HNSCC patients has not markedly improved in recent decades [2]. The poor survival is caused by locoregional recurrence, distant metastasis and second primary cancers. Many studies have indicated that cell adhesion and extra-cellular matrix proteins contribute to the cancer cell abilities for invasion, migration and metastasis [29]. However, the molecular mechanisms of invasion and metastasis of HNSCC have not yet been fully elucidated at present.

Growing body of evidence indicated that miRNAs contribute to cancer initiation, development and metastasis [6, 30]. Based on this point, we have sequentially identified tumor suppressive miRNAs and miRNA-mediated molecular targets contributing to cancer cell invasion and metastasis, such as *miR-1* regulates *TAGLN2*, *miR-145*-*FSCN1*/*LASP1*, *miR-133a*-*MSN* and *miR-138*-*VIM* [9, 20, 31-33]. It is known that these genes are categorized to actin-cytoskeleton or focal adhesion pathways, and deeply affect cell migration and invasion. Therefore, identification of miRNA-regulated focal adhesion pathway is important for further developments in human cancer research. Thus, we have been investigating how tumor suppressive miRNA regulates cancer pathways that are contributing to cancer cell migration and invasion.

Our previous study of HNSCC miRNA signatures showed that *miR-218* was downregulated in cancer tissues, suggesting that it would be a candidate of tumor suppressive miRNA [7, 8]. In this study, we validated the downregulation of *miR-218* in HNSCC clinical specimens. In human genome, two *miR-218* precursor genes, *miR-218-1* and *miR-218-2*, have identical sequences in the mature miRNA and mapped to human chromosome 4p15.31 and 6q35.1, respectively. Interestingly, the genomic regions of *miR-218-1* and *miR-218-2* are located in the introns of *SLIT2* and *SLIT3* genes respectively. The downregulation of *miR-218* in cancer cell was caused by promoter hypermethylation of *SLIT2* and *SLIT3* genes [22]. Silencing of *miR-218* by DNA hypermethylation was also reported in oral SCC using a function-based screening approach [21]. Little is known about epigenetic control of miRNAs expression in cancer cells. It will be important problems to elucidate it in cancer research fields in future.

Importantly, we found significant inhibition of cell migration and invasion in HNSCC cell lines (FaDu and SAS) by restoration of *miR-218*. These data suggested that *miR-218* had a tumor suppressive function especially contributing to cell migration and invasion in HNSCC. Tumor suppressive function of *miR-218* was also reported in several types of cancer targeting several oncogenic genes, such as *RICTOR* (oral cancer), *survivin* and *ROBO1* (nasopharyngeal cancer), and *ROBO1* (gastric cancer) [21, 22, 26]. A single miRNA is capable of targeting a number of genes to regulate biological processes globally. The elucidation of novel cancer pathways regulated by tumor suppressive *miR-218* is important for our understanding of HNSCC invasion and metastasis. Based on this view, we searched *miR-218*-mediated molecular pathways in HNSCC cells by combination of genome-wide gene expression data and *in silico* analysis. In this study, we focused on focal adhesion pathway and laminin-332 as a candidate target of *miR-218* in HNSCC cells. We chose laminin-332 for the following reasons. First, all components of laminin-332 (*LAMA3*, *LAMB3* and *LAMC2*) are listed on the candidate target genes in the focal adhesion pathway. Second, *LAMB3* have a putative *miR-218* target site in their 3'untranslated regions. Our data demonstrated that mRNA expression levels of three components of laminin-332 (*LAMA3*, *LAMB3* and *LAMC2*) were reduced by *miR-218* transfection in HNSCC cells. Downregulation of *LAMB3* by *miR-218* was also reported in cervical carcinoma cells by conducting qRT-PCR and western blot analyses [34]. In this study, we firstly demonstrated that *LAMB3* possessed an actual *miR-218* biding site by the luciferase reporter assay. These results showed that *miR-218* specifically targeted *LAMB3* in HNSCC cells. The functional significance of *LAMB3* in HNSCC was also investigated using a loss-of-function assay. Our data clearly showed that *LAMB3* functioned as an oncogene and strongly contributed to cancer cell migration and invasion.

Laminin-332, a heterotrimer composed of three chains (LAMA3, LAMB3 and LAMC2), is an adhesion substrate for epithelial cells, and regulates epithelial cell migration during epithelial regeneration and repair processes [35, 36]. Several immunohistochemical studies have shown that laminin-332 or its subunit LAMC2 is expressed in tumor cells at the invasion front or in budding tumor cells in many types of human cancers such as adenocarcinomas of colon, breast, pancreas and lung and SCC of esophagus, and melanoma [35]. Therefore, one of the chains, LAMC2 is a specific marker for invasive tumors [37]. The beta-catenin (Wnt) signaling pathway is known to induce a coordinate expression of laminin-332-LAMC2 chain and MT1-MMP in colorectal carcinomas [38]. In this study, *LAMC2* was upregulated in cancer tissues and confirmed previous reports. Although there is no putative *miR-218* target site in *LAMC2* gene, silencing of *LAMC2* mRNA expression was observed in *miR-218* transfectants. Because *LAMC2* was also downregulated in the expression profile of si-*LAMB3* transfectants, it is assumed that the downregulation of *LAMB3* somehow suppresses *LAMC2* expression. The analysis of the molecular mechanism of downregulation of *LAMC2* by *miR-218* is needed.

Furthermore, we asked why *LAMB3* has an oncogenic function in HNSCC. We conducted gene expression analysis using si-*LAMB3* to monitor *LAMB3*-controlled genes. In this study, several integrin signaling downstream molecules (*JUN*, *RAC1* and *PXN*) were downregulated in the expression profile of si-*LAMB3* transfectants. Many studies indicated that laminin-332 binds to several cell-surface receptors, such as integrins, epidermal growth factor receptor and syndecan-1 [39-41]. Among them, the integrins are cell surface transmembrane proteins that mediate the extracellular signals and the intracellular pathways leading to cell cycle controlling, cell migration and invasion of cancer cells [42].

Integrins are heterodimeric transmembrane receptors composed of an alpha and a beta subunit. To date, a total of 18 different alpha and 8 different beta subunits have been identified, accounting for at least 24 distinct integrin heterodimers [43]. Among those integrins, laminin-332 interacts with two major integrins receptors, alpha3beta1 and alpha6beta4, promoting the formation of focal adhesions and stable anchoring contacts [36]. In cancer cells, laminin-332-integrin alpha6beta4 interaction triggers a number of signaling cascades, promoting both cell migration and cancer survival [44]. Very interestingly, integrin beta4 gene (*ITGB4*) was reduced in *miR-218* transfectant in our profile and *ITGB4* contained putative *miR-218* binding site. We confirmed that *ITGB4* was reduced by *miR-218* transfection by PCR methods (data not shown). These data suggested that *miR-218* regulated laminin-332-integrin alpha6beta4 signal pathway in HNSCC cells. Silencing of tumor suppressive *miR-218* and upregulation of its signal pathway directly contribute to cancer cell migration and invasion in HNSCC.

In conclusions, our analysis data showed tumor suppressive *miR-218* contributed to cancer cell migration and invasion through regulating focal adhesion pathway, especially laminin-332. Elucidation of tumor suppressive *miR-218*-regulated cancer pathways provides the novel therapeutic strategy to control local tumor recurrence and distant metastasis of HNSCC.

## METHODS

### HNSCC cell lines

Two human HNSCC cell lines were utilized: FaDu (derived from hypopharyngeal squamous cell carcinoma) and SAS (derived from a primary lesion of tongue squamous cell carcinoma). FaDu and SAS were cultured in DMEM with 10% FBS in a humidified 5% CO_2_ atmosphere at 37°C.

### RNA isolation

Total RNA was isolated using TRIzol Reagent (Invitrogen, USA) according to the manufacturer's protocol. RNA concentrations were determined spectrophotometrically, and molecular integrity was checked by gel electrophoresis. RNA quality was confirmed using an Agilent 2100 Bioanalyzer (Agilent Technologies, USA).

### Quantitative reverse-transcription-PCR (qRT-PCR)

First-strand cDNA was synthesized from 1.0 μg of total RNA using a High Capacity cDNA Reverse Transcription Kit (Applied Biosystems). Gene-specific PCR products were assayed continuously using a 7900 HT Real-Time PCR System according to the manufacturer's protocol. TaqMan® probes and primers for *LAMA3* (P/N: Hs00165042_m1), *LAMB3* (P/N: Hs00165078_m1), *LAMC2* (P/N: Hs01043711_m1) and *GUSB* (P/N: Hs99999908_m1) (the internal control) were obtained from Applied Biosystems (Assay-On-Demand Gene Expression Products). The expression level of *miR-218* (Assay ID: 000521) was analyzed by TaqMan quantitative real-time PCR (TaqMan® MicroRNA Assay, Applied Biosystems) and normalized to *RNU48* (Assay ID: 001006). The relative expression levels were analyzed using the 2^−ΔΔCT^ method [45]. All reactions were performed in triplicate, and included negative control reactions that lacked cDNA.

### Mature miRNA transfection and small interfering RNA treatment

The following RNA species were used in this study: mature miRNA, Pre-miR™ miRNA Precursor (hsa-*miR-218*; Applied Biosystems, USA, P/N: AM17100), negative control miRNA (Applied Biosystems, P/N: AM17111), small interfering RNA (Silencer Select, Applied Biosystems, si-*LAMB3*, P/N: s8075 and s8076) and negative control siRNA (Stealth™ RNAi Negative Control Medium GC Duplex, Invitrogen, P/N:12935-300). miRNAs were incubated with Opti-MEM (Invitrogen) and Lipofectamine™ RNAiMax Reagent (Invitrogen) as described previously [12]. Transfection efficiency of Pre-miR™ in cell lines was confirmed based on downregulation of *TWF1* (*PTK9*) mRNA following transfection with miR-1 as previously reported [9, 31].

### Cell proliferation, migration and invasion assays

Cells were transfected with 10 nM miRNA by reverse transfection and 3 × 10^3^ cells were transferred to each well of a 96-well plate. After 72 h, cell proliferation was determined with the XTT assay, using the Cell Proliferation Kit II (Roche Molecular Biochemicals, Germany) as previously reported [7, 9].

A cell migration assay was performed using modified Boyden Chambers (Transwells, Corning/Costar #3422, USA) containing an uncoated Transwell polycarbonate membrane filter with 8 μm pores in 24-well tissue culture plates. Cells were transfected with 10 nM miRNA by reverse transfection and plated in 10 cm dishes at 8 × 10^5^ cells. After 48 h, 2 × 10^5^ cells were added to the upper chamber of each migration well and were allowed to migrate for 48 h. After gentle removal of the nonmigratory cells from the filter surface of the upper chamber, the cells that migrated to the lower side were fixed and stained with Diff-Quick (Sysmex Corporation, Japan). The number of cells migrating to the lower surface was determined microscopically by counting four areas of constant size per well.

A cell invasion assay was carried out using modified Boyden chambers containing Transwell-precoated Matrigel membrane filter inserts with 8 μm pores in 24-well tissue culture plates at 2 × 10^5^ cells per well (BD Biosciences, USA) [31]. All experiments were performed in triplicate.

### Target gene search for miR-218 and pathway analysis

A genome-wide screen was performed to identify gene targets for *miR-218* in FaDu and SAS cells. SurePrint G3 Human GE 8×60K Microarray (Agilent Technologies) was used for expression profiling of *miR-218* transfectants in comparison with miRNA-negative control transfectants. miRNA-control transfectants that produced raw signal values of less than 1,000 were excluded before comparisons were made. miRWalk March 2011 release [http://www.umm.uni-heidelberg.de/apps/zmf/mirwalk/] and TargetScan release 6.2 [http://www.targetscan.org/] were used to identify predicted target genes and their miRNA binding site seed regions. Sequences of the predicted mature miRNAs were confirmed using miRBase release 18.0 [http://microrna.sanger.ac.uk/].

### Clinical HNSCC specimens

Written consent for tissue donation for research purposes was obtained from each patient before tissue collection. The protocol was approved by the Institutional Review Board of Chiba University. Thirty-five pairs of primary tumor tissues and corresponding normal epithelial tissues were obtained from patients with HNSCC in Chiba University Hospital (Chiba, Japan) from 2007 to 2012. The normal tissue was confirmed to be free of cancer cells by pathologic examination. The specimens were immersed in RNAlater (Qiagen, USA) and stored at −20°C until RNA was extracted. The patients were classified according to 2002 Union for International Cancer Control TNM staging criteria before treatment.

### Western blot analysis

Cells were harvested and lysed 48 h after transfection. Each cell lysate (50 μg of protein) was separated using Mini-PROTEAN TGX gels (Bio-Rad, USA) and transferred to PVDF membranes. Immunoblotting was performed with polynoclonal LAMA3 antibody (sc-20143; Santa Cruz, USA), polyclonal LAMB3 antibody (HPA008069; Sigma-Aldrich, USA) and monoclonal LAMC2 antibody (MAB19562; Millipore, USA). GAPDH antibody (ab8245; Abcam, UK) was used as an internal control. The membrane was washed and incubated with anti-rabbit IgG, HRP linked antibody or anti- mouse IgG, HRP linked antibody (#7074 and #7076; Cell Signaling Technology, USA). Complexes were visualized with an Immun-Star™ WesternC Chemiluminescence Kit (Bio-Rad), and the expression levels of these proteins were evaluated by ImageJ software (ver.1.44; http://rsbweb.nih.gov/ij/index.html).

### Plasmid construction and dual-luciferase reporter assays

Partial sequences of the LAMB3 3' untranslated region that contains miR-218 target site

(ggcatgccattgaaactaagagctctcaagtcaaggaagct
gggctgggcagtatcccccgcctttagttctccactgggg
aggaatcctggaccaagcacaaaaacttaacaaaagtgatgta
aaaatgaaaagccaaataaaatctttggaaaagagcctggaggttc)

were inserted between the XhoI and PmeI restriction sites in the 3'UTR of the hRluc gene in the psiCHECK-2 vector (Promega, USA). FaDu and SAS were then transfected with five ng vector, 10 nM mature miRNA molecules, Pre-miRNA miR-218 (Applied Biosystems), and one μL Lipofectamine 2000 (Invitrogen) in 100 μL Opti-MEM. Firefly and Renilla luciferase activities in cell lysates were determined using a dual-luciferase assay system (E1910; Promega). Normalized data were calculated as the quotient of Renilla/firefly luciferase activities.

### Statistical analysis

The relationships between two groups and the numerical values obtained by qRT-PCR were analyzed using the Mann-Whitney U test or the paired t-test. The relationship among more than three variables and numerical values was analyzed using the Bonferroni adjusted Mann-Whitney U test. All analyses were performed using Expert StatView (version 4, SAS Institute Inc., USA).

## Supplementary Tables


